# Spontaneous Coronary Artery Dissection Beyond the Typical Demographic: Multivessel Involvement in a Male Patient

**DOI:** 10.7759/cureus.98558

**Published:** 2025-12-06

**Authors:** Kiran Sheikh, Ali Hameed

**Affiliations:** 1 Cardiology, William Harvey Hospital, Kent, GBR; 2 Cardiology, Mayo Hospital, Lahore, PAK

**Keywords:** male patient, multivessel scad, non-atherosclerotic myocardial infarction, scad in men, spontaneous coronary artery dissection

## Abstract

Spontaneous coronary artery dissection (SCAD) is an uncommon but increasingly recognized cause of acute myocardial infarction, predominantly affecting younger women without traditional atherosclerotic risk factors. The underlying pathophysiology remains incompletely understood and is thought to involve a complex interplay between arterial wall vulnerability and precipitating stressors. Moreover, challenges persist in achieving timely diagnosis and defining optimal management strategies. Occurrence of SCAD in men is particularly rare, often leading to diagnostic uncertainty and potential delays in appropriate management.

We report the case of a 43-year-old man with a history of hypertension and obesity who presented with a non-ST-elevation myocardial infarction (NSTEMI). Given his risk profile, an atherosclerotic aetiology was initially presumed. However, coronary angiography demonstrated distal left anterior descending (LAD) artery tapering with an appearance that was thought to be consistent with SCAD. Owing to his atypical demographic for SCAD, this angiographic impression generated a diagnostic dilemma, prompting further evaluation with cardiac magnetic resonance imaging (CMR), which revealed a multivessel infarction pattern. An interval repeat angiogram later demonstrated evolving features of healing SCAD in both the LAD artery and right coronary artery, confirming the diagnosis of multivessel SCAD. Although intracoronary imaging would have been ideal to enhance diagnostic clarity, it was not performed due to the distal location of the suspected SCAD lesions and variable operator experience with intracoronary imaging.

This case underscores the importance of considering SCAD even in atypical demographics such as male patients, where preconceived diagnostic assumptions may delay recognition. Greater awareness of its variable angiographic appearance and clinical patterns can help reduce diagnostic uncertainty and improve timely management.

## Introduction

Spontaneous coronary artery dissection (SCAD) predominantly affects younger or middle-aged women, accounting for 87-95% of cases in most series [1]. This striking female predominance has been partly attributed to hormonal influences increasing vascular vulnerability, supported by its higher incidence during pregnancy and the postpartum period; the exact mechanisms behind this female predominance remain unclear. Conversely, men represent only around 10% of SCAD cases [1]. They tend to present at a younger age and are more often associated with physical stressors rather than the emotional triggers typically described in women. Importantly, there appears to be no significant difference in clinical outcomes between the genders. However, a reversed gender bias exists - even when angiographic or clinical features are suggestive, SCAD is often under-recognized in men, potentially delaying diagnosis and management [1]. Early recognition is therefore essential, as misdiagnosis may lead to inappropriate interventions and worse outcomes. 

## Case presentation

A 43-year-old male patient with a past medical history of hypertension and obstructive sleep apnoea presented with the sudden onset of severe retrosternal chest pain while working in a restaurant at 8 a.m. The pain was intense, associated with sweating and mild shortness of breath, and lasted approximately 45 minutes before gradually settling. He was a non-smoker, had no history of dyslipidaemia, and denied any family history of premature cardiovascular disease.

**Figure 1:**
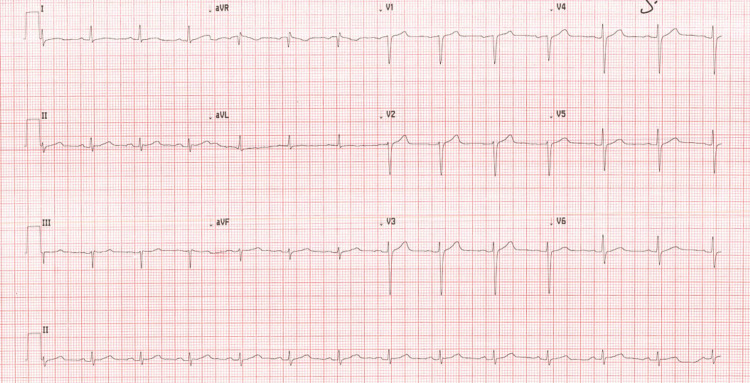
Electrocardiogram on admission showing normal intervals and no ischemic changes

On examination, he was obese with a body mass index of 40 kg/m^2^ (normal range: 18.5 to 24.9 kg/m^2^), blood pressure of 150/100 mmHg (normal value: <120/80 mmHg), and a heart rate of 80 beats per minute (normal range: 60 to 100 bpm). Cardiovascular and respiratory examinations were otherwise unremarkable. His initial electrocardiogram (ECG) performed in the emergency department showed sinus rhythm with no evidence of ischaemia or abnormal intervals (Figure 1).

High-sensitivity troponin I taken two hours after the onset of pain, was elevated at 688 ng/L (reference range: 0-34 ng/L) and rose further to 4938 ng/L six hours after the pain onset. His lipid profile revealed a total cholesterol of 7.3 mmol/L (normal value: <5 mmol/L) and a low-density lipoprotein (LDL) cholesterol of 5.13 mmol/L (normal value: <3 mmol/L), while HbA1c was 38 mmol/mol (normal value: <42 mmol/mol). He was diagnosed with a non-ST elevation myocardial infarction (NSTEMI) and commenced on dual antiplatelet therapy, low molecular weight heparin, a high-intensity statin, and bisoprolol.

**Figure 2:**
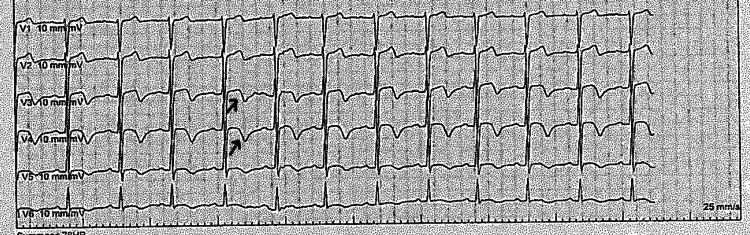
Electrocardiogram showing biphasic T waves in the anterior leads Arrows pointing to biphasic T waves in leads V3 and V4.

A transthoracic echocardiogram performed on the second day of admission showed a borderline low left ventricular systolic function with an estimated ejection fraction of 54 ± 5% by Simpson’s biplane method, but no regional wall motion abnormalities. The right ventricle and both atria were normal in size and function, and no valvular pathology was identified. An ECG, performed while he was pain free, showed biphasic T wave inversions in the anterior leads (Figure 2). 

A coronary angiogram was performed on the same day. The right coronary artery (RCA) appeared patent but ectatic, with incomplete opacification of the distal vessels due to the large calibre (Video 1).

**Video 1:** First angiogram showing ectatic right coronary artery (RCA) with poor opacification of the distal branches

The left anterior descending (LAD) artery, arising from the left coronary cusp, showed distal vessel tapering (Video 2).

**Video 2:** First angiogram showing the distal left anterior descending (LAD) artery tapering with an appearance consistent with type II SCAD. Arrow showing segment of distal LAD artery which has narrowed down with no evidence of intracoronary thrombus. Type II spontaneous coronary artery dissection (SCAD) is defined as diffuse narrowing of varying length and severity [2].

The left circumflex artery (LCx) arose anomalously from the right coronary cusp and appeared unobstructed. No left main stem was identified. The distal LAD artery appearance was quite typical for type II SCAD (defined as diffuse narrowing of varying severity and length [2]) with no obvious intraluminal thrombus or atherosclerotic plaque. However, due to atypical scenario, we decided to get an inpatient cardiac magnetic resonance imaging (CMR) to confirm diagnosis.

**Figure 3:**
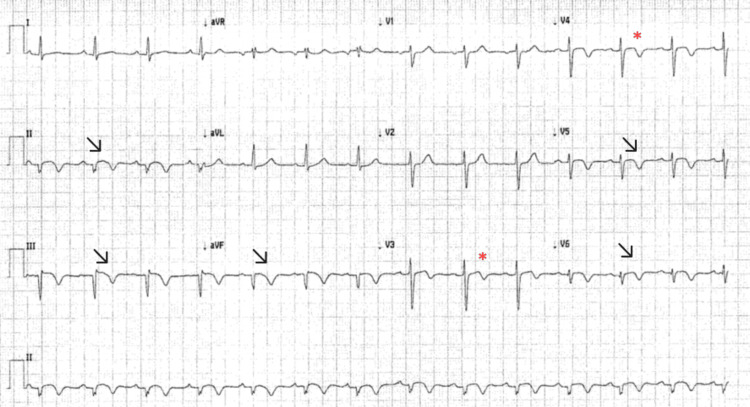
Repeat ECG showed settling ST segments in the inferior and lateral leads with T wave inversions Arrows show settling ST segments and T wave inversion in II, III, avf, V5 and V6 leads. Asterisks show previous biphasic T waves in V3 and V4.

While awaiting the CMR, the patient was kept under observation and was monitored with daily ECGs and troponin measurements. On day seven, his ECG showed some ST segments changes with T wave inversions in the inferior lateral leads (Figure 3) and his troponin level was 4689 ng/L.

A cardiac magnetic resonance imaging (MRI) performed the same day revealed a low-normal left ventricular end-diastolic volume with mildly reduced ejection fraction (55%) and mild septal left ventricular hypertrophy. There was myocardial oedema involving the mid inferior, inferolateral, and apical inferior walls, as well as near-transmural infarction in these regions, with evidence of microvascular obstruction. Additionally, small focal infarctions were noted in the distal LAD artery territory, including the apical anterior wall and apical septum, along with a small inducible perfusion defect in the mid inferoseptum. These findings were interpreted as being consistent with an acute or recent myocardial infarction in the mid-to-distal RCA or LCx territory, with smaller infarctions in the distal LAD artery territory, possibly representing embolic or SCAD-related events.

A bubble echocardiogram was performed to exclude a patent foramen ovale, which was negative. The case was discussed with the regional SCAD multidisciplinary team. It was agreed that appearance of distal LAD artery might pass for type II SCAD [2], but RCA images were sub optimal, and the cardiac MRI findings remained inconclusive between multivessel SCAD and atherosclerotic/embolic acute coronary syndrome (ACS). The recommendation was to repeat coronary angiography and consider intracoronary imaging for further clarification. It was also advised to continue treating him on the lines of atherosclerotic ACS.

The patient was discharged on day 19 before this review was finalised. He was re-admitted on day 21 for further evaluation. By this time, troponin levels had become negative. A repeat coronary angiogram with an intent to perform intra-coronary imaging was done on day 24. It demonstrated typical angiographic appearances of type II SCAD [2], involving the distal LAD artery (video 3) and the posterior descending artery (PDA) (video 4).

**Video 3:** Interval angiogram showed partially healed distal left anterior descending (LAD) artery SCAD SCAD: Spontaneous coronary artery dissection; An arrow pointing to the segment of the distal LAD artery, which has partially healed with some residual tapering.

**Video 4:** Interval angiogram showing right coronary artery (RCA) with sudden tapering of posterior descending artery (PDA) typical of type II SCAD SCAD: Spontaneous coronary artery dissection; An arrow points to segment of posterior descending artery (PDA) with distal tapering. Type II SCAD is diffuse narrowing of varying length and severity [2].

The angiographic appearance on this interval angiogram was so convincing that operator did not perform intra-coronary imaging and also because the lesions were too distal. The patient was managed conservatively and discharged home with a plan for follow-up in the specialist SCAD clinic.

In summary, the patient’s initial presentation with chest pain, elevated cardiac biomarkers, and unremarkable ECG findings led to a diagnosis of NSTEMI. Despite the initial presumed aetiology of atherosclerotic coronary disease, the series of imaging studies, including the interval angiogram, finally confirmed multivessel SCAD involving both the LAD artery and RCA territories. All the events from admission to final discharge are shown on the event timeline (Table 1).

**Table 1:** Event timeline CMRI: Cardiac Magnetic Resonance Imaging; ECG: Electrocardiogram; LAD: Left Anterior Descending; PDA: Posterior Descending Artery; RCA: Right Coronary Artery; SCAD: Spontaneous Coronary Artery Dissection. Type II SCAD mentioned in the last row is defined as diffuse narrowing of varying severity and length [2].

Day	Event	Key findings/Results
0	Admission with chest pain	ECG normal, Troponin 688 ng/L up trending to 4938 ng/L
1	Stable, chest pain free	Troponin down trending to 1791 ng/L
2	First angiogram	ECG: biphasic T waves in anterior leads. Angiogram: distal LAD artery tapering, ectatic RCA
3	Recurrence of mild chest pain	No new ECG changes
7	Stable, chest pain free	ST elevation (II, III, aVF, V5 and V6) without chest pain. Troponin 4689 ng/L
9	Evolution of changes in Inferior leads with T wave inversion	CMRI: multivessel infarctions, SCAD vs embolic
19	Discharged	Stable
21	Re-admission to confirm diagnosis	Troponin negative
24	Repeat angiogram and discharged	Type II SCAD in distal LAD artery & PDA

## Discussion

SCAD accounts for approximately 1-4% of all cases of ACS; however, its true prevalence remains uncertain because of under-recognition and diagnostic limitations [2]. Missed or delayed diagnoses often result from low clinical suspicion in younger patients, limited awareness among healthcare professionals, and challenges in angiographic interpretation. Even when SCAD is considered, it is most frequently suspected in younger female patients without conventional cardiovascular risk factors [2].

Pathologically, SCAD represents the formation of a false lumen within the epicardial coronary arterial wall, caused by separation of the intima or the intima-media complex from the adventitia [3]. Two main mechanisms have been proposed: an “inside-out” mechanism, where an intimal tear allows blood to enter the vessel wall, and an “outside-in” mechanism, where spontaneous rupture of the vasa vasorum causes an intramural haematoma without an intimal break. Both processes compress the true lumen and precipitate myocardial ischaemia, distinguishing SCAD from atherosclerotic ACS, in which plaque rupture and thrombosis are the key events. Traumatic and iatrogenic dissections are excluded from this definition.

The “typical” SCAD patient is a woman in her 40s or 50s with few or no traditional risk factors for coronary artery disease [1]. Women account for 87-95% of cases, with a mean age of 44-53 years [1]. The preponderance of SCAD among women and its association with pregnancy, hormonal therapy, and infertility treatment have led to speculation about a hormonal contribution, although this relationship remains incompletely understood [4].

In contrast, SCAD in men is uncommon and poorly characterised, accounting for only about 10% of reported cases [2]. Male patients tend to present at a younger age and are more likely to report intense physical exertion or isometric activity as a precipitating factor, whereas emotional stressors are more often identified in women [5]. Some studies have suggested a higher recurrence rate in men [6].

Multivessel SCAD is seen in 9-23% of the cases, so it is a relatively rare presentation [2]. Data from the Spanish SCAD registry suggested that no significant differences in short- or long-term major adverse cardiovascular events were observed in patients with multivessel SCAD [7].

Angiographically, SCAD is classified into three main patterns [2]. Type I (≈25% of cases) demonstrates multiple radiolucent lumens with contrast staining. Type II, the most common, presents as diffuse smooth narrowing with either distal normalisation or extension to small terminal branches. Type III mimics focal atherosclerosis and typically requires intravascular imaging (Optical Coherence Tomography (OCT) and Intravascular Ultrasound (IVUS)) for confirmation [2]. Although these angiographic types appear distinct in theory, real-world differentiation can be difficult, particularly in the presence of overlapping features or poor contrast filling [2]. The presence of coronary tortuosity and the absence of intraluminal thrombus often favour SCAD [8].

Despite improved understanding of SCAD pathophysiology over the last decade, diagnostic challenges persist, especially in atypical populations. Misclassification as atherosclerotic disease remains frequent, and the term “SCAD mimic” has emerged to describe angiographically similar but pathogenetically distinct entities [2]. These mimics, such as coronary embolism, vasospasm, myocarditis, or recanalized thrombus, require careful differentiation because their management differs fundamentally. While intracoronary imaging can significantly enhance diagnostic accuracy, its usefulness is limited by lesion location and the fact that these techniques are highly operator-dependent, factors that may preclude their safe or effective use in real world practice [2]. 

When intracoronary imaging is not feasible, such as when the lesion is distal or involves small branches, non-invasive imaging can provide valuable complementary information [8]. CMR can help exclude myocarditis or Takotsubo-like cardiomyopathy, and identify infarction patterns consistent with SCAD [8]. However, when CMR shows transmural infarction, distinguishing SCAD from thromboembolic events may remain challenging. In such cases, interval imaging demonstrating healing or vessel remodelling is often required to confirm the diagnosis [4].

This case contributes to the growing evidence that SCAD can occur in men and may involve multiple vessels. It underscores the importance of maintaining a high index of suspicion for SCAD even in patients outside the “typical” demographic profile. Greater awareness and careful use of multimodality imaging including interval imaging can help reduce diagnostic uncertainty and ensure appropriate management.

## Conclusions

This case highlights the diagnostic challenges of SCAD in male patients, a group in which the condition remains exceedingly rare and often under-recognised. Awareness of its variable angiographic appearances is crucial, as even when features are pathognomonic, diagnostic uncertainty may persist due to preconceived assumptions and overlapping features. This can result in delays in diagnosis and management, with confirmation frequently requiring interval imaging. Incorporating guidance on such atypical presentations and imaging-based follow-up strategies into future clinical guidelines could help improve early recognition and optimise patient outcomes.
